# Medical students’ experiences of their own professional development during three clinical terms: a prospective follow-up study

**DOI:** 10.1186/s12909-017-0886-3

**Published:** 2017-02-27

**Authors:** Susanne Kalén, Hanna Lachmann, Maria Varttinen, Riitta Möller, Tomas S. Bexelius, Sari Ponzer

**Affiliations:** 1 Dept of Clinical Science and Education, Södersjukhuset, Karolinska Institutet, Stockholm, Sweden; 2 Sophiahemmet University, Stockholm, Sweden; 3 Dept of Medical Epidemiology and Biostatistics, Karolinska Institutet, Stockholm, Sweden

**Keywords:** Medical students, Professional development, CanMEDS, Academic emotions, Contextual activity sampling

## Abstract

**Background:**

A modern competency-based medical education is well implemented globally, but less is known about how the included learning activities contribute to medical students’ professional development. The aim of this study was to explore Swedish medical students’ perceptions of the offered learning activities and their experiences of how these activities were connected to their professional development as defined by the CanMEDS framework.

**Methods:**

A prospective mixed method questionnaire study during three terms (internal medicine, scientific project, and surgery) in which data were collected by using contextual activity sampling system, i.e., the students were sent a questionnaire via their mobile phones every third week. All 136 medical students in the 6th of 11 terms in the autumn of 2012 were invited to participate. Seventy-four students (54%) filled in all of the required questionnaires (4 per term) for inclusion, the total number of questionnaires being 1335. The questionnaires focused on the students’ experiences of learning activities, especially in relation to the CanMEDS Roles, collaboration with others and emotions (positive, negative, optimal experiences, i.e., “flow”) related to the studies. The quantitative data was analysed statistically and, for the open-ended questions, manifest inductive content analysis was used.

**Results:**

Three of the CanMEDs Roles, Medical Expert, Scholar, and Communicator, were most frequently reported while the four others, e.g., the role Health Advocate, were less common. Collaboration with students from other professions was most usual during the 8th term. Positive emotions and experience of “flow” were most often reported during clinical learning activities while the scientific project term was connected with more negative emotions.

**Conclusions:**

Our results showed that it is possible, even during clinical courses, to visualise the different areas of professional competence defined in the curriculum and connect these competences to the actual learning activities. Students halfway through their medical education considered the most important learning activities for their professional development to be connected with the Roles of Medical Expert, Scholar, and Communicator. Given that each of the CanMEDS Roles is at least moderately important during undergraduate medical education, the entire spectrum of the Roles should be emphasised and developed during the clinical years.

**Electronic supplementary material:**

The online version of this article (doi:10.1186/s12909-017-0886-3) contains supplementary material, which is available to authorized users.

## Background

One often used model for defining the competences of a physician is the CanMEDS framework which describes the abilities required to meet health care needs [1]. These abilities are expressed as seven Roles, i.e., Medical Expert, Communicator, Collaborator, Manager, Health Advocate, Scholar, and Professional, which together build up the professional competence of a physician (Additional file 1). The CanMEDS framework [1] has been used in the undergraduate medical education programme at Karolinska Institutet, Sweden, where this study was conducted. During their studies, medical students participate in a longitudinal mentoring programme in which they assess their own professional development according to the CanMEDS Roles [2, 3]. In that work, and also in this study, professional development was defined as development of the physician’s different Roles in the CanMEDS Framework [1]. Kalén et al. [3] found that longitudinal and formalised mentoring promoted the students’ understanding of the “wholeness” of the professional competence of a physician. It also helped them to integrate themselves as individuals with their future professional role.

The CanMEDS framework [1] also emphasises that such aspects as collaboration and communication with patients, colleagues, and other professions are as important as medical knowledge and skills in patient care. Furthermore, the WHO report [4] on interprofessional education (IPE) stresses the importance of interprofessional collaboration for patient safety. It is also known that emotions play an important role in both interprofessional communication and learning in general. Positive emotions, such as enthusiasm, have been related to a feeling of “flow,” i.e., when an optimal challenge is combined with optimal competence [5].

Competence-based medical education has been well implemented globally, but some still argue that more focus should be placed on the development of professional identity and not only on the specific competences included in the role of a competent physician [6]. Therefore, it is of interest to investigate medical students’ experiences of a competence-based curriculum and how they relate different learning activities to their professional development, specifically during the clinical courses.

Assessment tools have been developed to assess medical students’ professional competence [7, 8] but as far as we know, there are no published studies that have followed their experiences of their own professional development over time.

Therefore, we followed continuously a group of medical students’ experiences of course activities related to CanMEDS Roles [1] during three terms by using contextual activity sampling methodology [9, 10]. To our knowledge, there are no previously published studies on professional development using this type of method where students report on their activities in close connection with the time when they occur.

The primary aim of this study was, therefore, to explore medical students’ perceptions of the offered learning activities and their experience of how these activities were connected with their professional development as defined by the CanMEDS Framework. The secondary aim was to investigate medical students’ interprofessional collaboration and also how their emotions (positive, negative, and optimal experiences, i.e., “flow”) were related to their studies.

## Methods

### Participants and study context

This is a mixed method study and part of a longitudinal study conducted during 2012–2014 at Karolinska Institutet, Sweden, with a focus on undergraduate medical students’ professional development. All medical students in the 6th term in the autumn of 2012 (*n* = 136) were eligible to participate. Ninety-eight of them (72%) agreed to participate and 74 (54%) filled in all of the required questionnaires (4 per term) for inclusion. Forty-nine of the participants (66%) were females and 38 (51%) were <27 years old. During the three terms of data collection, the students attended the following courses: Internal Medicine (6th term), Scholarly Scientific Project (7th term), and Surgery (8th term), including 2 weeks on an interprofessional training ward [11]. In Sweden, the undergraduate medical education programme consists of 11 terms (corresponding to 5.5 years). The first four terms are mainly preclinical and, from term 5, almost all of the educational activities are conducted in clinical environments. During all 11 terms, students are in continuous contact with the same mentor and they also participate in various mandatory activities related to their professional development, including ethical discussions. One term (the 7th) is dedicated to a scientific research project that can be done in a clinical or preclinical environment and results in a scientific report, written independently by the student.

### Procedure for data collection

To facilitate self-reported data gathering, the Contextual Activity Sampling System (CASS) methodology was chosen [9]. The CASS methodology is inspired by ideas stemming from the Experience Sampling Method [12], which make it possible to collect longitudinal and frequent qualitative and quantitative data concerning ongoing activities and emotions in a specific context [10, 13].

The students were asked to respond every third week to a CASS questionnaire via their mobile phone during terms 6–8 (a total of 19 questionnaires). One questionnaire took approximately 3 to 5 min to complete and included a total of 15 questions (Additional file 2). A text message was sent to remind the students to respond to the questionnaire within one week.

### CASS questionnaires

First, the students were asked to specify the course they had been engaged in during the last 3 weeks. The questions focused on their experience of their learning activities in relation to the CanMEDS Roles and collaboration during that period. Six questions concerning both positive and negative emotions (the PANAS scale; [14]) were used and they were also asked to rate their own experienced competence and challenges regarding their current learning activity on a seven-point Likert scale (1 corresponding to strongly disagree and 7 to strongly agree) [15]. Responses to these two combined questions indicate the level of optimal experience (“flow”) [12, 16, 17]. Optimal experience is described as a situation in which the learners feel that the ongoing activities are challenging and meaningful and that they also have the competence to manage them [16, 17].

Three types of questions were used in the questionnaires: (a) questions with free text answers, for example: “Which learning activities during the last 3 weeks were most important for your professional development?”; (b) multiple-choice questions, with stated alternatives, e.g., “Indicate the two CanMEDS Roles that your last 3 weeks of learning activities were mainly related to.” They were asked to indicate *two* Roles since the Role “Medical Expert” is sometimes considered to integrate all the other Roles [1] and, therefore, there was a risk that, otherwise, they would only state that role for all the times. The third type of question (c) included rankings (7-point Likert scale, 1 indicating the minimum, 7 the maximum), e.g., positive emotions (interest, enthusiasm, and resoluteness) and negative emotions (irritation, anxiety, and nervousness).

### Statistical procedures

The Statistical Package for Social Sciences (SPSS version 22; SPSS Inc., Chicago, IL, USA) and a spreadsheet application (Excel) were used for the statistical data analysis. Since each participating student answered the CASS questions more than once, standardised scores (Z-scores) for rated experiences of academic emotions were calculated. The Z-scores were standardised for each question and for each student by setting the mean to 0 and the SD to 1. This was done in order to reduce the effects of variances related to individual answering tendencies [18]. To assess whether there were any variations and significant correlations between the investigated perceptions in relation to CanMEDS, the particular course, the learning activities, collaboration, and term, an ANOVA and an *X* ^2^ test (chi-squared test) was performed. A *p* value of <0.05 was regarded as statistically significant. Competence rated ≥ 5, in combination with challenge rated ≥ 5, was estimated as experience of “flow” [17].

### Content analyses of the qualitative data

Qualitative data from free text answers concerning learning activities were categorised to a further analysis together with quantitative data. Data were analysed manually using inductive manifest content analysis [19–21]. Meaning units from the answers (in total, 1390 statements) were identified and sorted into 51 subcategories by MV, SK, and HL. Eleven of the categories were excluded because their contents were not relevant to the research subject. Subcategories were then grouped into seven descriptive categories (Table 1). Subcategories and categories were frequently discussed by all researchers in the group during the process until a consensus was reached. Qualitative data from other free text answers were used for base-line information.

**Table 1:** Overview of the identified categories and underlying subcategories; the categories describe the most important activities for learning during the 3 weeks prior to responding to the questionnaire; the categories are related to three domains: knowledge, skills, and attitudes

Subcategories	Categories	Domain
Studying, work on project, examination, laboratory task, presenting to others, writing, seminar, self-assessment, reading articles	Teaching and learning about theory – active student	Knowledge
Introduction, lecture, traditional teaching, subject knowledge	Teaching and learning about theory – not an obviously active student	
Clinical training, simulation, handling clinical situations, independence, own responsibility, participation in the clinical team, clinical assessment, interviewing	Learning in the clinical environment – active student	Skills
Clinical practice, clinical placement, auscultation	Learning in the clinical environment – not an obviously active student	
Supervision, conversation with mentor/supervisor, developing a professional attitude, ethical discussion, professional development, feedback from supervisor/teacher	Communication and collaboration with professionals	Attitudes
Conversation with patient/relative, conversation with patient/relative in clinical situations	Communication and collaboration with patients and/or their relatives	
Reflection with peers, meeting course mates, the work shop, focusing on professional development, meeting like-minded	Communication and collaboration with peers or like-minded	

### Ethical considerations

The students were informed, orally and in writing, about the study’s aims and design. They were also informed that the results would only be used for research purposes and that participation was voluntary and without any impact on course marking. This study was approved by the Regional Ethical Review Board, Karolinska Institutet, Stockholm, Sweden (Dnr: 2012/1227-32).

## Results

The 74 students included in this study completed a total of 1335 questionnaires, 413 of which were derived from term 6, 480 from term 7, and 442 from term 8, thus resulting in an acceptably equal distribution of responses during the terms. Most of the questionnaires were filled in at home (54%), which was to be the most usual place for reflection. Other places where the questionnaires were filled in were the clinical department (9%), on the move (7%), and at the library (5%). Fifteen per cent of the students answered “Other place.”

### Most important learning activities

Analyses of free text data concerning the most important learning activities resulted in seven categories (Table 1). These categories were related to knowledge, skills and attitudes, levels of student activity, and whether the learning activities were conducted in the presence of patients, staff, peers, or others. The categories presented are based on their manifest content, along with illustrative quotes.

### Knowledge

#### Teaching and learning about theory – active student

This category comprises activities and learning situations related to theoretical knowledge and teaching methods where the students had an active role, such as seminars, writing of scientific texts, examinations, reading articles, laboratory tasks, and collecting data for the research project or to present to others.

“I am now working on extraction of data from medical records. Reading medical records provides, in a way, opportunities for professional development…”

#### Teaching and learning about theory – not an obviously active student

This category comprises situations and learning activities related to theoretical knowledge and “traditional” teaching methods whereby the students had more of a passive role, such as lectures, specific subject knowledge, e.g., infectious diseases or introduction to a course.

“I have learned statistics and other useful things.”

### Skills

#### *Learning in the clinical environment* – *active student*

This category comprises situations and learning activities related to clinical skills in the clinical environment where the student had an active role such as making x-ray assessments, simulator training, analysing biopsy specimens, leading the round at the hospital, or doing his/her first sutures. The learning activity occurred in or without the presence of a patient.

“Participated in an out-patient clinic and was in the operating theatre at the orthopaedic department.”

#### *Learning in the clinical environment* - *not an obviously active student*

This category comprises situations and learning activities related to clinical environments or environments connected with the clinic where the student’s activity was not obvious, such as clinical placements, auscultations or just practice. The learning activity was in or without the presence of a patient.

“I followed a physician who informed a patient’s relative about the very poor prognosis for the patient who had suffered a major stroke. It was very instructive since the physician had a very nuanced and professional attitude to the relative and took his time to answer questions and acted like a fellow human being.”

### Attitudes

#### Communication and collaboration with professionals

This category comprises activities and learning situations that include communication and collaboration with such professionals as physicians, mentors, supervisors, and researchers. Their communication and collaboration included feedback, ethical discussions, and professional attitudes and behaviour.

“… an ethical discussion with a physician who has been working for many years.”

#### Communication and collaboration with patients and/or their relatives

This category comprises situations and learning activities in clinical environments, which include communication and collaboration with patients and patients’ relatives on clinical wards and such activities as delivering messages about health status.

“…talking to a relative of a very sick patient.”

#### Communication and collaboration with peers or like-minded persons

This category comprises situations and learning activities which include communication and collaboration with other medical students, course mates, or like-minded persons. This category also includes reflection together with peers.

“…to critique a classmate’s research project report at the half-time seminar.”

### Learning activities related to CanMEDS Roles

The number of categorised learning activities during each term (6, 7, and 8) was combined with the CanMEDS Roles reported at the same time (Table 2). Both CanMEDS Roles reported each time were included. The Roles of Medical Expert, Scholar, and Communicator were most usual (highlighted with bold letters in Table 2). The role of Medical Expert was most usual in term 6 (*n* = 130, 43% of all reported activities) and term 8 (*n* = 127, 39% of all reported activities) in combination with learning in the clinical environment (an active student and a not obviously active student). The role of scholar was reported most often in term 7 (*n* = 178, 59% of all reported activities) in combination with teaching and learning about theory – active student. The role of communicator was reported most often in term 7 (*n* = 94, 65% of all reported activities) in combination with teaching and learning about theory – active student. In general, the Roles of Collaborator, Manager, and Professional were reported less frequently and the Role of Health Advocate was least reported regardless of the learning activities during all of the three terms.

**Table 2:** The number of categorised learning activities (row), during each term (6, 7, and 8), combined with the CanMEDS Roles (column) reported at the same time

Categorised learning activities	CanMEDS Roles																				
	Medical Expert			Communicator			Collaborator			Scholar			Health Advocate			Manager			Professional		
Teaching and learning about theory - active student	49	8	26	13	**94**	3	7	32	5	34	**178**	17	11	1	5	5	63	2	13	28	7
Teaching and learning about theory - not an obviously active student	19	4	29	3	5	2	5	2	5	13	12	12	2	0	1	1	6	2	2	0	15
Learning in the clinical environment - active student	51	2	**127**	12	5	24	11	3	52	29	8	47	7	0	6	4	5	10	12	1	24
Learning in the clinical environment - not an obviously active student	**130**	4	98	26	17	22	15	6	24	76	34	38	14	2	3	6	12	6	36	3	25
Communication and collaboration with professionals	16	0	16	2	17	5	1	9	4	11	59	6	4	0	1	0	36	3	8	2	3
Communication and collaboration with patients and/or their relatives	30	1	24	17	4	7	3	3	10	14	3	15	4	0	2	2	3	0	13	0	6
Communication and collaboration with peers or like mimded	9	0	4	2	2	2	1	1	0	7	8	1	0	0	0	0	4	0	1	0	1
Term	6	7	8	6	7	8	6	7	8	6	7	8	6	7	8	6	7	8	6	7	8

The most frequently reported CanMEDS Roles within each category are highlighted with bold type

### Collaboration

**Fig. 1:**
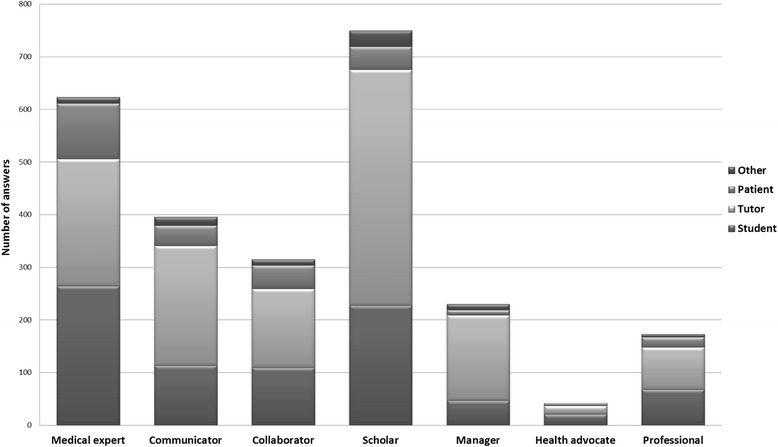
Number of students reporting that they collaborated with someone (students, tutors, patients or other) in combination with the reported CanMEDS Roles at the same time

**Fig. 2:**
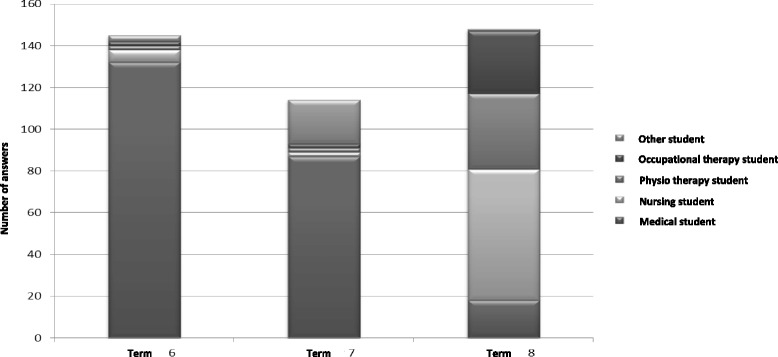
Number of students reporting collaboration with medical students, nursing students, physiotherapist students, occupational therapy students and students from some other profession during terms 6, 7, and 8

Collaboration was reported most often when the learning activities were related to the CanMEDS Roles of Medical Expert, and Scholar. The students mainly reported collaboration with other students and tutors and less often with patients and others, e.g., patients’ relatives (Fig. 1). They collaborated significantly more often (*p* < 0.05) with other students in terms 6 (*n* = 138) and 8 (*n* = 193) than in term 7 (*n* = 96). Interprofessional collaboration with students from other educational programmes (nursing, physiotherapist, occupational therapist or other programmes) was significantly (*p* < 0.05) higher in term 8 than in terms 6 and 7, while in terms 6 and 7, they mostly collaborated with other medical students (Fig. 2).

### Positive and negative emotions

**Fig. 3:**
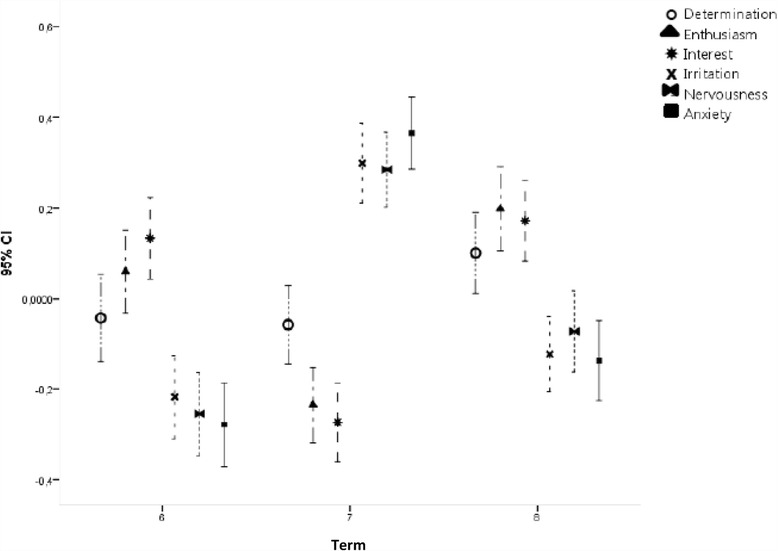
Variation in positive and negative emotions experienced by medical students between the investigated terms

The students’ emotions connected with learning activities varied between the three terms. The experience of being interested and enthusiastic was significantly more frequent (*p* < 0.05) in terms 6 and 8 than in term 7. The feelings of irritation, nervousness, and anxiety, i.e., negative emotions, were significantly more frequent (*p* < 0.05) in term 7 than in terms 6 and 8. The feeling of resoluteness was highest in term 8; however, this emotion did not vary between the terms to the same extent as the other emotions (Fig. 3).

### Optimal experience – “flow”

**Fig. 4:**
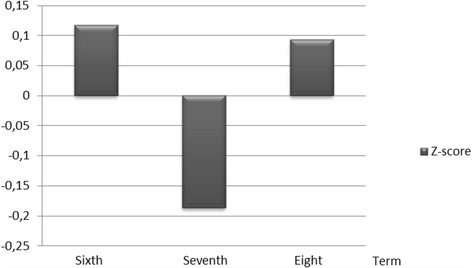
Variation in optimal experience (“flow”) during terms 6, 7, and 8 (Z-score)

The students’ experiences of “flow” differed somewhat between the three terms. Experience of flow was reported most rarely (20%) during term 7 when the students were working on their scientific projects (Fig. 4). During clinical activities in terms 6 and 8, the experience of flow was 24%. The variation in flow during and between terms is visualised in Fig. 4.

## Discussion

In this study we explored medical students’ experiences of their professional development by studying the connection between learning activities and CanMEDS Roles. Our results showed that the most usual areas of professional development were the Roles of Medical Expert and Scholar. Students clearly related the Role of Medical Expert to learning activities in the clinical environment while they related both the role of Communicator and the role of Scholar to teaching and learning about theory. Learning activities in the clinical environment in term 6 seemed to be less student-activating than clinical learning activities in term 8. An unanticipated finding was that they related the role of Communicator with teaching and learning about theory, and that this role was more often reported during the research project course than during their clinical placements. Furthermore, enthusiasm, interest, and “flow” were highest when their learning occurred in the clinical environment, whereas irritation, nervousness, and anxiety, as well as a lack of flow were most usual during individual research projects.

In order to focus on the students’ professional development, we chose to use the CanMEDS framework [1] since this model is well known globally and is also used in our undergraduate medical programme [2, 3]. The students were asked to state the *two* Roles that they felt were related to their learning activities since there was an obvious risk that if they were asked to state just one, they might have answered “Medical Expert,” the role that might overarch all other Roles [1]. One of our main findings was that three of the CanMEDS Roles, i.e., Medical Expert, Scholar, and Communicator, were the most prominent, whereas the four others, i.e., Collaborator, Manager, Health Advocate, and Professional, were noted less frequently. It is possible that the meaning of these Roles was not clear to the students, for which reason they were reported less often, a finding in line with prior research in this area [2, 3]. It might also have been difficult for the students to associate these Roles with their ongoing clinical learning activities if they were not familiar with all the Roles in the CanMEDS Framework. They seemed to pay more attention to learning about theoretical knowledge and clinical skills than practicing communication and collaboration, which are at least as important competences for becoming a professional physician. Educators should pay more attention to the area of attitudes in order to enhance the students’ awareness and development of these competences. Student-activating assignments appeared to be most important for the students’ professional development. Thus, educators should use student-activating pedagogy more often in the clinical environment, including communication and collaboration with professionals, patients, and peers. That would make the students more aware of the importance of developing these competences during clinical terms.

Surprisingly, the role of communicator was connected most often with term 7, during which the students worked on their individual scientific projects, and not with clinical terms when they actually met patients and communicated with them, their relatives, other professions and supervisors on a daily basis. One explanation might be that during the research project course, the students were required to give several scientific presentations of their own, in which they were to critically review fellow-students’ presentations and projects and discuss the findings in their groups. The students also have coordinated teaching in scientific communication during that term, including criteria for evaluating written and oral communications. Nevertheless, these results suggest that the students did not perceive interaction with patients, staff, and clinical supervisors as “real” communication. Nevertheless, the physician-patient communication and the interprofessional team communication are central parts of the daily clinical work and should be actively supported by supervisors and teachers. It is also important that the students get feedback on their communication skills in the clinical environment as it is well know that communication is the area where most mistakes related to patient safety occur [22].

Collaboration with students from other professions was reported significantly more often during term 8. This finding was not surprising and was probably related to the fact that all students have to attend a mandatory 2-week interprofessional training ward (IPTW) course [11] during term 8. Even though the IPTW course is relatively short, it might have an impact on the students’ communication behaviours by highlighting the importance of, and the influence on, the possibility of collaboration with other students, which is also stressed in the WHO report [4] on interprofessional education (IPE). An interesting finding was that even if the students reported higher scores on “collaboration with other students” in term 8, they did not connect their learning activities with the CanMEDS role of collaborator. This points out the importance of the fact that also the supervisors have to highlight all the roles and competences of a professional physician when supervising medical students.

Positive and negative emotions [5] varied between the terms. Positive emotions were reported most often in terms 6 and 8 when the activities were connected with learning in clinical environments. On the other hand, negative emotions were reported most often in term 7 when the learning activities were connected with teaching and learning about theory, i.e., scientific project work. The reported experience of flow followed the same pattern, i.e., it was reported most often in terms 6 and 8. This was not surprising since flow is a positive state of mind with flexible and creative learning strategies that facilitate positive emotions, while more rigid strategies and procedures entail negative emotions [5].

According to Marton and Booth [23] and Mayer [24], it is important to strive for learning activities that improve meaningful learning, which is a deeper level of learning and understanding. In a study on nursing students’ learning on a clinical training ward, it was found that important factors for meaningful learning were the experience of belongingness in the care team, the mutual relationship with patients, and the experience of authenticity [25]. One might ask if the learning activities during clinical placements in terms 6 and 8 offered meaningful learning with experiences of belongingness and opportunities to create a mutual relationship with patients, inasmuch as the role of Medical Expert was the one reported most often and those of Communicator and Collaborator were reported less often, although the students were obliged to report two Roles. On the other hand, it is possible that the students’ interpretation of the different Roles varied so that they included the other Roles in the Role of Medical Expert.

The learning activities during term 7 (when the students conducted their scientific project) were different compared to activities during the other terms. The students had to follow a regulated research process, but, at the same time, work rather independently and with self-discipline in order to maintain their timelines. These issues may explain the students’ negative emotions and lack of “flow” since they were dependent on their supervisors to succeed, but were still responsible for doing the work on their own. This finding is in line with previous research showing that supervision is one of the most important factors for successful project work [26]. Even though the scientific project work was a less positive experience, it gave the students the opportunity to develop other competences, such as the role of communicator, which is needed for their professional development.

The strengths of this study were the large number of student responses, continuously collected in the students’ learning context and also the rather even distribution of responses over the terms. The CASS methodology enabled data collection in a continuous manner and therefore presented a novel way to follow the students’ experiences as compared to traditional post-course questionnaires [27]. One weakness might be that the students reported their activities themselves, which may have resulted in inaccuracies caused by recall bias regarding the frequency of certain competences. Furthermore, we asked the students to report only on two Roles and to rate the most important activity for learning. The possibility of reporting on several roles and activities may have enriched the data. Another strength is that the coding and the definition of categories have been discussed by all the authors until a consensus was reached. Rich description was used to enable transferability to other settings.

## Conclusion

Our results showed that it is possible, even during clinical courses, to visualise the different areas of professional competence defined in the curriculum and to connect these competences with the actual learning activities. Students halfway through their medical education considered the most important learning activities for their professional development to be connected with the Roles of Medical Expert, Scholar, and Communicator. Given that each of the CanMEDS Roles is at least moderately important during undergraduate medical education, the entire spectrum of the Roles should be emphasised by educators when developing the curriculum. Further studies are needed to explore whether there are other factors, such as personalities and learning styles, that impact on students’ professional development.

## Additional files

Additional file 1: Definition of the CanMEDS roles. Definition of the CanMEDS roles which together build up the professional competence of a physician. (DOCX 16 kb)
Additional file 2: Questionnaire for medical students. The CASS questionnaire the students were asked to respond every third week. (DOC 83 kb)

## Abbreviations

CASS: Contextual activity sampling system
IPE: Interprofessional education
IPTW: Interprofessional training ward
PANAS: Positive and negative affect schedule
