# Supplementary material for: Medical students’ experiences of their own professional development during three clinical terms: a prospective follow-up study
Source: BMC Med Educ. 2017 Feb 27;17:47. doi: 10.1186/s12909-017-0886-3 (PMC5327532; doi:10.1186/s12909-017-0886-3)
Supplement: Additional file 1: — Definition of the CanMEDS roles. Definition of the CanMEDS roles which together build up the professional competence of a physician. (DOCX 16 kb) [file 12909_2017_886_MOESM1_ESM.docx]

**Appendix 1**

**Definition of the CanMEDS roles^[[1]](#footnote-1)^**

**Medical Expert**

Definition

As Medical Experts, physicians integrate all of the CanMEDS Roles, applying medical knowledge, clinical skills, and professional values in their provision of high-quality and safe patient-centred care. Medical Expert is the central physician Role in the CanMEDS Framework and defines the physician’s clinical scope of practice.

# **Communicator**

## Definition

As Communicators, physicians form relationships with patients and their families* that facilitate the gathering and sharing of essential information for effective health care.

# **Collaborator**

## Definition

As Collaborators, physicians work effectively with other health care professionals to provide safe, high-quality, patient-centred care.

# **Leader**

## Definition

As Leaders, physicians engage with others to contribute to a vision of a high-quality health care system and take responsibility for the delivery of excellent patient care through their activities as clinicians, administrators, scholars, or teachers.

# **Health Advocate**

## Definition

As Health Advocates, physicians contribute their expertise and influence as they work with communities or patient populations to improve health. They work with those they serve to determine and understand needs, speak on behalf of others when required, and support the mobilization of resources to effect change.

# **Scholar**

## Definition

As Scholars, physicians demonstrate a lifelong commitment to excellence in practice through continuous learning and by teaching others, evaluating evidence, and contributing to scholarship.

# **Professional**

## Definition

As Professionals, physicians are committed to the health and well-being of individual patients and society through ethical practice, high personal standards of behaviour, accountability to the profession and society, physician-led regulation, and maintenance of personal health.

1. <http://www.royalcollege.ca/rcsite/canmeds/canmeds-framework-e> 2016-11-06 [↑](#footnote-ref-1)
