# Supplementary material for: Medical students’ experiences of their own professional development during three clinical terms: a prospective follow-up study
Source: BMC Med Educ. 2017 Feb 27;17:47. doi: 10.1186/s12909-017-0886-3 (PMC5327532; doi:10.1186/s12909-017-0886-3)
Supplement: Additional file 2: — Questionnaire for medical students. The CASS questionnaire the students were asked to respond every third week. (DOC 83 kb) [file 12909_2017_886_MOESM2_ESM.doc]

**Appendix 2.**

**Questionnaires for medical students**

Hi, now it is time to fill in a questionnaire by clicking on the link [**www.xxxxxx.se**](http://www.xxxxxx.se/)The questionnaire is available for a week.

Thank you for your co-operation!

Yours truly, PU Research Group

Female   Male Born, year: 19__ __

Which course and which part of that course do you participate at this moment?..................................

Where are you right now?

- Lecture Theater
- Seminar room
- Group room
- Clinical department
- Library
- At home
- On your way to some place
- Other place, specify………

Describe what you are doing right now?............................................................................................

Which competences of a future physician does your ongoing learning (course?) activity relate to?
Select the two main competences.

- Medical expert
- Communicator
- Collaborator
- Scholar
- Manager
- Health Advocate
- Professional
- Other competences, specify

Do you collaborate with anyone in this part of the course: No Yes

If so, with whom do you collaborate? You can choose one or several alternatives

- Another student
- Supervisor
- Patient
- Other

If you collaborate with another student, to which educational programme does this student belong?

- Medicine
- Nursing
- Physiotherapy
- Occupational therapy
- Student from another educational program, specify……………..

How do you feel about the ongoing course activity at this moment?

***Very little Very much*** **1 2 3 4 5 6 7**

How challenged do you feel?

How competent do you feel?

Below are some words that describe different kinds of moods and feelings. Please rate the words according to how you feel at this moment (currently?).

Very Little Very much **1 2 3 4 5 6 7**

Determined

Enthusiastic

Interested

Irritated

Nervous

Worried

Which learning activity did you find to be most important for your professional development during the last three weeks? Describe…………………..

Thanks for answering the questions!

Here comes your adlibris value code. (30 SEK)

Every third week during term time, you will receive a new questionnaire.

Yours truly, PU Research Group
